# Reliability and Validity of the SHFT Running Power Meter

**DOI:** 10.3390/s21227516

**Published:** 2021-11-12

**Authors:** Jesper Emil Linkis, Thomas Christian Bonne, Jacob Bejder, Esben Krogh Rasmussen, Andreas Breenfeldt Andersen, Nikolai Baastrup Nordsborg

**Affiliations:** Department of Nutrition, Exercise and Sports (NEXS), Faculty of Science, University of Copenhagen, Nørre Allé 51, DK-2200 Copenhagen, Denmark; Linkis@nexs.ku.dk (J.E.L.); tbonne@nexs.ku.dk (T.C.B.); jbr@nexs.ku.dk (J.B.); krogh@nexs.ku.dk (E.K.R.); anan@nexs.ku.dk (A.B.A.)

**Keywords:** sports technology, sensors, wearable, running, exercise, training, testing, fitness device, gadget

## Abstract

The SHFT device is a novel running wearable consisting of two pods connected to your smartphone issuing several running metrics based on accelerometer and gyroscope technology. The purpose of this study was to investigate the reliability and validity of the power output (PO) metric produced by the SHFT device. To assess reliability, 12 men ran on an outdoor track at 10.5 km·h^−1^ and 12 km·h^−1^ on two consecutive days. To assess validity, oxygen uptake (VO_2_) and SHFT data from eight men and seven women were collected during incremental submaximal running tests on an indoor treadmill on one to four separate days (34 tests in total). SHFT reliability on the outdoor track was strong with coefficients of variance (CV) of 1.8% and 2.4% for 10.5 and 12 km·h^−1^, respectively. We observed a very strong linear relationship between PO and VO_2_ (r^2^ = 0.54) within subjects, and a very strong linear relationship within each subject within each treadmill test (r^2^ = 0.80). We conclude that SHFT provides a reliable running power estimate and that a very strong relationship between SHFT-Power and metabolic rate exists, which places SHFT as one of the leading commercially available running power meters.

## 1. Introduction

**Figure 1:**
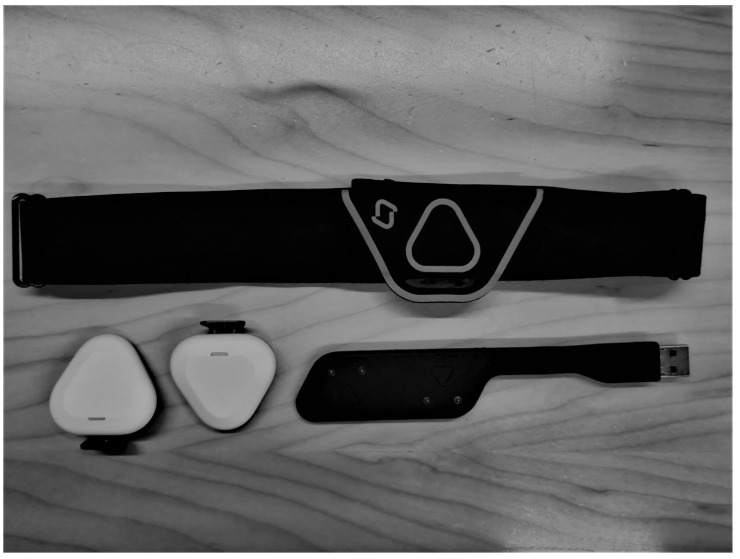
Components of the SHFT Device: A chest band, two pods, and a USB charger.

Running distances of ≥800 m seems to be predominated by the aerobic energy system [1,2]. Aerobic running performance is determined by maximal oxygen uptake (VO_2max_), running economy, and the lactate threshold [3]. Following specific types of training regimes, increases in VO_2max_ [4,5,6,7,8], running economy [9,10,11,12], and velocity at lactate threshold [13,14,15] are expected. Monitoring these parameters requires costly laboratory equipment and the applicability of velocity at, e.g., lactate threshold is limited by external factors such as slope, surface, and wind resistance. Power output (PO) determination during running is a classical challenge in exercise physiology [16]. As opposed to the widely applied PO measurements in cycling, there is no simple way to quantify a runner’s PO, e.g., by use of an ergometer. Thus, measures of center of gravity displacement and/or estimated cost of limb movements have been applied for decades with highly varying results and no possibility for consumer usage [16]. However, recent developments in wearable sports devices have resulted in several approaches to provide runners with an easily obtainable quantification of PO. Currently, at least seven running power meters are commercially available: SHFT [17], Stryd [18], RunScribe [19], Garmin Running Power [20], Polar Grit X/Vantage [21], RPM_2_ [22], and FeetMe Sport [23]. Only a few studies have investigated the reliability and validity of these devices [24,25,26,27,28]. Garmin Running Power and Polar Grit X/Vantage uses barometer and GPS data, while RPM_2_ and FeetMe Sport uses pressure and motion sensors in the shoe soles to estimate running power. SHFT, RunScribe, and Stryd all use accelerometers attached to the shoe to estimate running power, and of these three, SHFT is the only device not yet scientifically investigated. The SHFT device (Figure 1) consists of two pods using accelerometer and gyroscope technology [29]. One is attached to the shoe and the other is attached to a chest band. The pods are paired with a smartphone using the official SHFT application. The device takes in 8000 readings per second and has an output of more than 10 metrics [17].

Reliability is usually investigated as the coefficient of variance in a test-retest approach, whereas validity is investigated as concurrent validity by correlating PO with oxygen uptake (VO_2_). Of the mentioned devices Stryd is the most investigated and PO of the Stryd device appear more accurate and reliable than devices from Garmin, RunScribe, and Polar [24] with a coefficient of variance of <5% [24,25]. Regarding validity, mixed results of the Stryd device exist with coefficients of determination (PO vs. VO_2_) ranging from 0.08 to 0.84 [24,26,28] and 0.36 for PO vs running economy [27].

Provision of a reliable and valid running PO holds high potential for giving immediate feedback on all levels of running intensity from slow efforts to brief sprinting and intermittent exercise which is not possible with the otherwise valuable heart rate measurements. Ideally, measured running PO should accurately reflect changes in external factors such as surface, slope, and wind resistance. Additionally, accurately determined running PO has a huge potential in optimizing training and racing for athletes as well as amateurs and recreational runners. However, it can be argued that a reliable measure even with low validity is of interest for runners since it provides the opportunity to monitor individual progress. 

The purpose of the present paper is to evaluate reliability and validity of the SHFT device PO estimate and secondary to evaluate reliability of measures that must be assumed to be reliably detected and of value for technical running analysis.

## 2. Materials and Methods

Data were collected on two occasions: (1) during submaximal running on an open 400 m track on two consecutive days and (2) during submaximal running on an indoor treadmill with simultaneous measurement of pulmonary gas exchange at fixed velocities. 

### 2.1. Outdoor Track Running

Twelve men (35.3 ± 11.3 years, 74.1 ± 8.0 kg, 178.8 ± 6.9 cm) participated in the outdoor track measurements aiming to evaluate the reliability of SHFT-sensors at two submaximal speeds. The SHFT device estimates several running variables (Table 1); however, the primary variable of interest in the current study was the estimation of PO. At two consecutive days, the subjects arrived at the same 400 m outdoor track, which conform to the standards of the International Association of Athletics Federations [30]. Subjects were asked to weigh themselves before attending and to enter their weight and height in their individual user profile in the SHFT application on their own smartphone. At arrival, the subjects were equipped with one SHFT sensor at the bottom lace of their right foot and one SHFT sensor attached to a chest band, which were connected to their smartphone. Each subject used the same sensors across test days. The subjects warmed up for 5–10 min before running 8 min at ~10.5 km·h^−1^ in a single file behind a pacer. After two minutes of rest, the subjects ran for 8 min at ~12 km·h^−1^ in the same manner. A national elite runner controlled the pace using a GPS-watch as well as lap timing. The estimated PO was calculated as a 2-min average when the GPS pace was visually steady and lap timing was closest to 137 and 120 s for pace 10.5 km·h^−1^ and 12 km·h^−1^, respectively. One result was excluded from the 10.5 km·h^−1^ retest due to the chest band of a subject sliding down towards the waist.

### 2.2. Indoor Treadmill 

**Figure 2:**
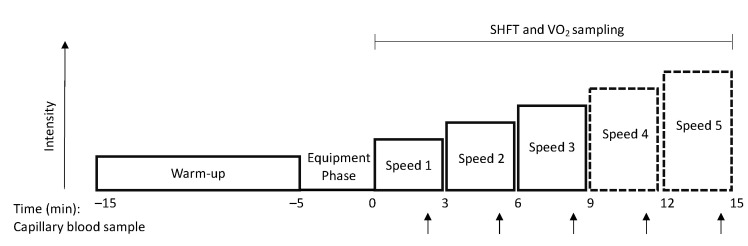
Schematic of the workflow in the indoor treadmill test. Solid boxes indicate speeds which were completed in all 34 tests, whereas speed 4 and speed 5 were completed in 24 tests and 1 test, respectively. Arrows indicate capillary blood sampling.

**Figure 3:**
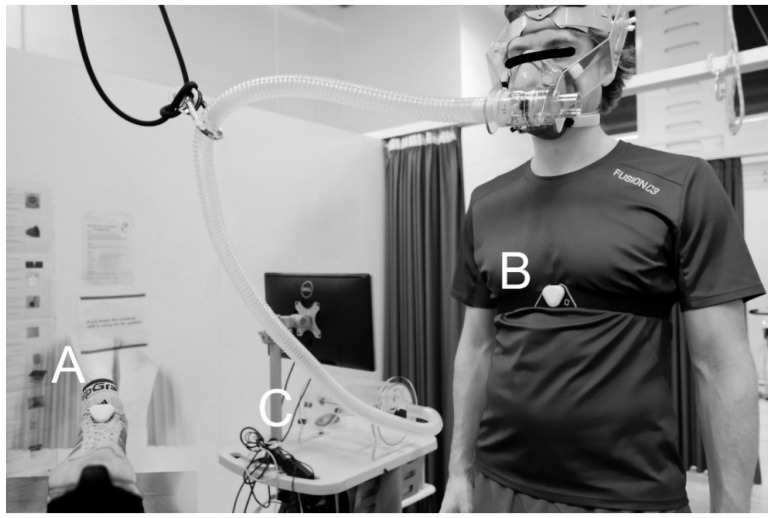
Demonstration of the indoor treadmill test setup where (**A**) illustrates a SHFT pod equipped on a random lace, (**B**) illustrates a SHFT pod equipped on a chest band, and (**C**) illustrates the mixing chamber positioned on a metabolic cart.

Eight men and seven women (26 ± 3 years, 66.3 ± 9.0 kg, 176 ± 10 cm and a maximal oxygen uptake of 57 ± 9 mL/kg/min) completed the indoor treadmill measurements 1–4 times separated by 1–5 weeks. Participants were tested on a treadmill (The Pro, Woodway USA, Inc., Waukesha, WI, USA) as illustrated in Figure 2 and Figure 3, starting with a 10 min warmup at the same absolute velocity across all test days, which was individualized for each subject. After the warm-up, the subjects were equipped with a SHFT-sensor (SHFT, Copenhagen, Denmark) on a random lace of the left or right foot and a SHFT-sensor attached to a chest band, which were connected to a smartphone using the SHFT application. Additionally, subjects were equipped with a mask connected to a mixing chamber for measuring pulmonary gas exchange of O_2_ and CO_2_ using an automated metabolic gas analysis system (Quark CPET, COSMED, Rome, Italy). Five minutes after the warm-up, the subjects initiated a submaximal running test with 3–5 speed increases of 1 km·h^−1^ every 3 min. The speed increase continued until capillary blood lactate values were >4 mmol·L^−1^, which was measured in the final minute of each speed level using an ABL 800 Flex (Radiometer, Brønshøj, Denmark). The coefficient of variance (CV) was calculated for all subjects who completed the indoor running test at least twice (n = 11) at two submaximal velocities. The coefficient of determination (r^2^) was calculated using all 34 tests for all subjects (n = 15). Oxygen uptake and SHFT data were calculated as an average of 30 s between time 1:10 and 1:40 at each speed.

### 2.3. Statistics

Statistical calculations were performed using the SPSS Statistical Software version 25 (SPSS Inc., Chicago, IL, USA). Data are presented as mean ± SD.

To evaluate the relationship between VO_2_ and estimated PO, the coefficient of determination was analyzed using a univariate general linear model [31] with PO as the dependent factor and VO_2_ as the covariate, while subject was considered a fixed factor. To consider a possible confounding factor of individual variation between timepoints a similar analysis with subject and time as fixed factors were carried out. Coefficients of determination (r^2^) were interpreted using Hopkins scale of magnitudes (www.sportsci.org (accessed on 30 August 2021)). However, as the scale is based on Pearson’s correlation coefficient, the scale was converted to values corresponding to the coefficient of determination. Thus, r^2^ < 0.01 is interpreted as trivial, 0.01–0.09 as small, 0.09–0.25 as moderate, 0.25–0.49 as strong, 0.49–0.81 as very strong, and >0.81 as nearly perfect. 

CV was calculated as the standard deviation of the differences between test days divided by the mean of all measures at the two test days for the respective variable. The outdoor track running CV was calculated using data from the two consecutive test days, whereas the CV for indoor treadmill data were calculated using the nearest two test days.

## 3. Results

### 3.1. Test Retest Reliability

#### 3.1.1. Power Output

On the indoor treadmill the mean velocity was 12.4 ± 1.4 km·h^−1^ and 13.4 ± 1.4 km·h^−1^ for speed 1 and speed 2, respectively. The mean difference in estimated PO between tests at speed 1 and speed 2 was 8.8 ± 8.7 and 9.9 ± 10.1 W, respectively. On the outdoor track, the mean difference between test days was 4.9 ± 3.5 and 7.4 ± 4.9 W at 10.5 km·h^−1^ and 12 km·h^−1^, respectively. The CV of the SHFT estimated PO for outdoor and indoor measures are presented in Table 1. 

#### 3.1.2. Secondary Metrics

The running efficiency had a mean difference between tests of 1.4% ± 2.1% at 10.5 km·h^−1^ and 1.6% ± 1.0% at 12 km·h^−1^ on the outdoor track. The mean difference in strides per minute at 10.5 km·h^−1^ and 12 km·h^−1^ was 1.8 ± 1.7 and 1.5 ± 1.7 strides·min^−1^, respectively. Other secondary metrics measured by the SHFT device and their test-retest reliability on the outdoor track running (best case) are presented in Table 1. 

### 3.2. Validity

**Figure 4:**
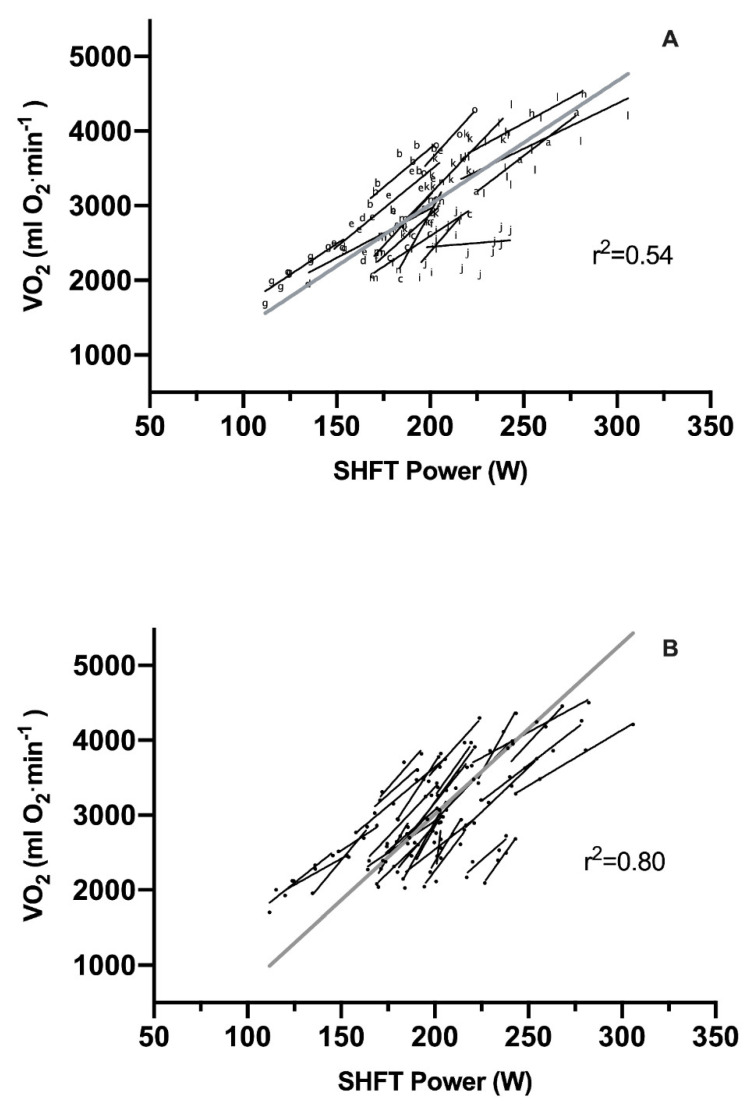
Linear regression analysis on oxygen uptake (VO_2_) and SHFT Power in watts (W). The gray lines represent the overall regressions. (**A**): Within subject regression. Subjects are denoted by alphabetical characters. Each black line represents the individual regression line of a subject. (**B**): Within subject and within time regression. Subjects are denoted by black circles. Each black line represents the individual regression line of a subject for each test day.

The relationship between the estimated PO and VO_2_ at different running speeds from the indoor treadmill measurements was analyzed within each subject providing a coefficient of determination (r^2^) of 0.54 (Figure 4A). When the analysis was adjusted to be within each subject at each timepoint the coefficient of determination was 0.80 (Figure 4B).

## 4. Discussion

This study evaluated the reliability and validity of running power output estimated by the SHFT device, as well as the reliability of parameters describing running technique. The SHFT device provides reliable estimation of PO with a low variation within each subject (CV < 5%). A very strong relationship was observed between oxygen uptake and estimated power output when analyzed within subject and within subject and time. 

### 4.1. Reliability 

In the present study, we demonstrate a test-retest CV of 1.8% at 10.5 km·h^−1^ and 2.4% at 12 km·h^−1^ for estimated PO by the SHFT device on an outdoor running track, but an inferior CV of ~5% using an indoor treadmill (Table 1). The difference may be due to inconsistencies in sensor equipping and potential differences in biomechanical running economy as the test-retests on the indoor treadmill were separated by up to five weeks, although we consider the latter unlikely. Other devices able to estimate running PO exist, but studies investigating their reliability and validity are few. One study compared five different tools to estimate running PO: Stryd App (connected to smartphone), Stryd Watch (connected to GPS-watch), Garmin Running Power, RunScribe, and Polar Vantage. During outdoor running, the Stryd App and Stryd Watch were considered the most reliable device to estimate running PO with a CV of 2.7% and 2.8% at 10 km·h^−1^ and 2.0% and 1.3% at 12 km·h^−1^, respectively [24]. Similarly, the Stryd power meter provides a CV of 4.5% during trail walking and 3.4% during trail running [25]. Notably, if the CV was calculated in the present study by the method of Cerezuela-Espejo et al. [24], a CV of 1.8% and 2.7% at 10.5 km·h^−1^ and 12 km·h^−1^, respectively, during outdoor running was evident with the CV during indoor treadmill running being 4.8% and 3.4% at speed 1 and speed 2, respectively. Thus, the SHFT device display a similar reliability compared to the currently most reliable device: the Stryd power meter.

### 4.2. Validity

Determining whether a running power meter estimates a valid PO can be done by examining the relationship between the PO estimate and VO_2_ at submaximal velocities since VO_2_ is dependent on and increases linearly with submaximal work rate [32]. In the present study the relationship between estimated PO and VO_2_ across test days was very strong (r^2^ = 0.54), but likely influenced by inconsistencies in sensor equipping (Figure 4A), which is supported by the higher coefficient of determination (r^2^ = 0.80) when the relationship was investigated within each test day (Figure 4B). Despite a very strong validity of the estimated PO, large differences in VO_2_ at, e.g., an intensity of 200 W (VO_2_ ~2000–3800 mL O_2_·min^−1^) between subjects exist. Although differences in running economy affect the VO_2_, it is unlikely to explain the entire dispersion. Rather, this suggests that the SHFT device has limitations for estimations of accurate PO across different running styles, although the dispersion is expected to be reduced by increased standardization of sensor equipping.

A very strong relationship between PO estimated by the Stryd device and VO2 exist (r^2^ > 0.82–87) [24,26], which was superior to the Garmin Running Power, RunScribe, and Polar Vantage [24]. However, in one study the regression analysis was analyzed as one sample and not within each subjects [24], which can yield highly misleading results [33]. Yet, the dispersion in their correlation do appear narrower (200 W equals ~2000–2400 mL O_2_·min^−1^ in the study by Cerezuela-Espejo et al., and 2000–3800 mL O_2_·min^−1^ in the present study). In contrast, the relationship between PO and running economy ranges between r^2^ = 0.04–0.64 for the Stryd device [27]. Others report a small (r^2^ = 0.08) relationship between PO/speed (W·kg^−1^/m·s^−1^) estimated by the Stryd device and VO_2_/speed (mL·min^−1^·kg^−1^/m·s^−1^) [28], but the methodology has been subject for criticism [34]. In summation, the current literature indicates that the SHFT device estimates a similar PO to that of the Stryd device, but the conflicting reports on current running power meter validity suggests that running power is yet to be perfected although more standardized research is warranted to determine validity.

### 4.3. Limitations 

In the current study, the comparison of reliability and validity of power output between the SHFT and other running devices relies on a comparison between the present results and results from similar studies on other running power output devices. Including additional devices for a direct comparison, preferably tested simultaneously, would have eliminated potential differences from the present to previous studies and strengthened the comparison of the SHFT device to other devices. However, this was not logistically feasible. Furthermore, a direct comparison between devices in different slope, wind or surface conditions would provide valuable insight to the usability of running power estimates. Another potential limitation in the present study is less controlled sensor equipping during indoor treadmill running, which may impair the reliability or validity. Finally, VO_2_ was determined 1:10–1:40 into each velocity, which may be inadequate to obtain steady state at the first velocity [35].

## 5. Conclusions

In conclusion, the power output metric of the SHFT device is a reliable tool with a low within-subject variation and a very strong relationship between metabolic rate and running power estimate, but differences in running style may affect the estimation of true running power. When comparing to previous studies evaluating running power estimates, it appears that SHFT is providing equally reliable and valid measures as the previous best performing device, the Stryd device. However, potential differences between studies limit the comparison. Future studies should confirm the reliability and validity of the SHFT device, preferably in different conditions and with a direct comparison to other power meters. Furthermore, validating the secondary metrics of the SHFT-device and of other running gadgets are a research area of interest. 

## Figures and Tables

**Table 1:** Reliability of SHFT metrics.

Indoor Treadmill	Speed 1 (n = 11)		Speed 2 (n = 11)	
	Mean	CV (%)	Mean	CV (%)
Power (W)	188.6	4.6	199.6	5.1
Outdoor Track	10.5 km/h (n = 11)		12 km/h (n = 12)	
Power (W)	191.3	1.8	207.9	2.4
Stride Rate (Stride·min^−1^)	163.1	2.2	166.8	1.0
Step Length (cm)	107.7	2.8	120.9	1.7
Landing (G)	−9.7	−22.3	−9.7	−20.1
Landing Angle (°)	−19.5	−20.0	−21.2	−15.4
Landing Position (Num)	5.3	17.1	5.7	12.6
Toe Off Angle (°)	47.4	9.2	49.0	14.5
Contact Time (ms)	303.2	3.1	290.1	4.5
Time in Air (ms)	420.4	4.1	427.8	3.9
Deceleration (G)	13.2	23.1	16.9	15.1
Body Bounce (cm)	6.2	1.8	6.2	1.2
Running Efficiency (%)	25.1	8.2	25.1	4.0
Running Effect (W)	47.7	6.8	51.7	5.9
Pace (km·h^−1^)	10.7	2.2	12.2	2.1

Mean values for power in watts (W) and the coefficient of variation (CV) for indoor treadmill and outdoor track running tests. For the outdoor track, mean values for the different SHFT metrics from the two testing days are also provided.

## Data Availability

Data is available upon request.
